# Distinct Haptic Cues Do Not Reduce Interference when Learning to Reach in Multiple Force Fields

**DOI:** 10.1371/journal.pone.0001990

**Published:** 2008-04-23

**Authors:** Nicholas Cothros, Jeremy Wong, Paul L. Gribble

**Affiliations:** 1 Department of Psychology, The University of Western Ontario, London, Canada; 2 Graduate Program in Neuroscience, The University of Western Ontario, London, Canada; 3 Department of Physiology and Pharmacology, The University of Western Ontario, London, Canada; University of Birmingham, United Kingdom

## Abstract

**Background:**

Previous studies of learning to adapt reaching movements in the presence of novel forces show that learning multiple force fields is prone to interference. Recently it has been suggested that force field learning may reflect learning to manipulate a novel object. Within this theoretical framework, interference in force field learning may be the result of static tactile or haptic cues associated with grasp, which fail to indicate changing dynamic conditions. The idea that different haptic cues (e.g. those associated with different grasped objects) signal motor requirements and promote the learning and retention of multiple motor skills has previously been unexplored in the context of force field learning.

**Methodology/Principle Findings:**

The present study tested the possibility that interference can be reduced when two different force fields are associated with differently shaped objects grasped in the hand. Human subjects were instructed to guide a cursor to targets while grasping a robotic manipulandum, which applied two opposing velocity-dependent curl fields to the hand. For one group of subjects the manipulandum was fitted with two different handles, one for each force field. No attenuation in interference was observed in these subjects relative to controls who used the same handle for both force fields.

**Conclusions/Significance:**

These results suggest that in the context of the present learning paradigm, haptic cues on their own are not sufficient to reduce interference and promote learning multiple force fields.

## Introduction

Motor skill learning is a remarkable feature of the primate nervous system. While humans are able to learn a large number of motor skills, how this is accomplished is poorly understood. To gain a better understanding of motor skill learning, researchers have explored how humans adapt to novel dynamics. Using robotic devices that apply forces to the hand (a force field) and perturb reach trajectories, previous studies have shown that subjects learn to precisely counteract the novel dynamics, thereby restoring normal movement [1]. This compensatory adjustment in motor output is termed “motor learning” and is thought to reflect an updating of neural representations of the physical properties of the motor effectors and the environment [1]–[6].

Adapting to novel dynamics is prone to interference, in which the learning of two different force fields is met with difficulty. Whereas subjects show proficient adaptation to a single force field, difficulties arise when subjects are confronted with a second, different force field [7]–[9]. Studies of interference frequently use the A1-B-A2 paradigm, which entails training subjects in an initial force field, followed by training in a second, different field, and finally retraining in the initial field. Interference is comprised of two distinct, detrimental effects on motor skill acquisition: proactive and retroactive interference. Adaptation to the first force field proactively interferes with adaptation to the second field. In addition, adaptation to the second force field retroactively interferes with retention of the initial field. This pattern has been demonstrated in a number of recent studies of motor learning using the A1-B-A2 paradigm [7]–[13], and interference in motor learning has been widely reported not only in the case of force field learning, but also in the case of learning perturbations of visual feedback [6], [8], [10], [13].

Interference in force field studies is a puzzling finding, as it seems to oppose humans' apparent facility in learning multiple motor skills. Recently it has been proposed that force field learning reflects learning the novel dynamics associated with a novel grasped object [14], [15]. This proposal followed the observation that force field learning does not generalize to arm movements in free space, in which grasp of the robotic device is released. The implication is that force field learning is not an updating of a single neural representation of movement dynamics, incorporating both the limb and the grasped object, but rather reflects the acquisition of a distinct neural representation of the dynamics of the grasped object [14]. Indeed, it has been proposed in previous theoretical models that cues associated with grasp aid in the acquisition of novel dynamics by providing distinct signals associated with motor tasks having different requirements [6].

In the present study we test the hypothesis that haptic cues associated with grasp facilitate the acquisition of multiple internal models of novel dynamics. Specifically, we test the possibility that interference in motor learning may be reduced when two different force fields are associated with differently shaped objects grasped in the hand. Subjects were instructed to guide a cursor to visually displayed targets while grasping a robotic manipulandum. The manipulandum applied two opposite force fields in accordance with the A1-B-A2 paradigm. Subjects were trained extensively in one force field, followed by training in the opposing field and retraining in the initial field. If haptic cues associated with grasp provide a contextual signal that facilitates the learning of multiple force fields, then interference ought to be reduced when each force field is associated with a unique grasp-related cue. It was found that the performance of subjects was not affected by changes in the shape of the grasped object and that interference persisted. These findings suggest that grasp-related cues alone are not sufficient for the learning of two different force fields.

## Methods

### Subjects

A total of 31 right-handed subjects between the ages of 18 and 22 years (mean = 18.5 years) participated in the present study. All subjects reported normal or corrected vision, no history of neurological, or musculoskeletal disorder and gave their written informed consent prior to participation. All procedures were approved by the University of Western Ontario Research Ethics Board.

### Apparatus

**Figure 1:**
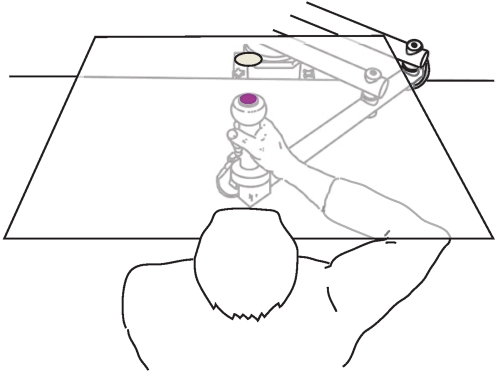
Experimental setup. Subjects grasped the handle of the InMotion robotic device (Interactive Motion Technologies, Cambridge, MA) with their right arm abducted at the shoulder and supported by a custom made air sled. Subjects produced horizontal-plane arm movements involving shoulder and elbow rotation to guide the motion of a cursor to a series of visual targets, projected using a computer controlled LCD projector onto a screen suspended 20 cm above the hand and reflected into view by a semi-silvered mirror positioned 10 cm below the screen. This created the illusion that the targets were positioned in the plane of the subject's arm movements.

Subjects grasped the handle of the InMotion^2^ robotic device (Interactive Motion Technologies, Cambridge, MA) with their right arm abducted at the shoulder and supported by a custom made air sled, which cushioned the upper arm with foam padding and produced a steady flow of air directed beneath the support system. The air sled allowed subjects to generate movements in a frictionless environment without fatiguing the arm [16]. Movement of the arm and the robot was restricted to a horizontal plane containing the shoulder (see Figure 1). Using movements of the arm, subjects guided the motion of a cursor to a series of targets which were projected using a computer controlled LCD projector onto a screen suspended 20 cm above the hand and reflected into view by a semi-silvered mirror positioned 10 cm below the screen. This created the illusion that the targets were positioned in the plane of the subject's arm movements.

The robot was programmed to apply forces to the hand during reaching movements to targets. Force magnitude varied with the velocity of the handle (and thus the hand). The direction of the applied forces was perpendicular to the direction of hand movement. The force fields were designed to perturb movement, creating curved reach trajectories. The direction of the forces perturbed movements in a counterclockwise (CCWFF) or clockwise (CWFF) direction. The force fields are described by the following equation:
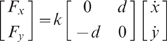
(1)where and are robot generated forces in the left/right and forward/backward direction, respectively, and and are hand velocities, k = 25 Ns/m, and d = +1.0 (CWFF) or d = −1.0 (CCWFF). Forces were zero at movement start and movement end when the robot was still and were maximal at peak hand tangential velocity. Force magnitude was at maximum at peak hand tangential velocity. Forces were controlled using custom software running under the RT Linux operating system on a Pentium 4 CPU. Robot handle positions, velocities and applied forces were sampled at 200 Hz and stored on a digital computer for analysis.

### Experimental Task

Subjects were instructed to move the cursor quickly, accurately and in a straight line towards the targets. Movements were made between a start target (corresponding to shoulder and elbow joint angles of 45 and 90 degrees) and three equidistant targets aligned on the circumference of a circle. Targets were 24 mm in diameter and were located 10 cm away from the start target. The middle target was located directly in front of the start target, and the left and right targets were located 30 deg to the left and right of the middle target, respectively. Subjects were asked to complete each movement within a timing window of 300–400 ms. Feedback about movement time was given on each trial by changing the color of the target, indicating that the movement was too slow, too fast, or completed within the appropriate window of time.

The manipulandum and the subject's arm were completely hidden from view and the experiments were run in darkness. Consequently, the subject was provided only with visual feedback of the position of the targets and the position of the cursor (and thus the hand), as well as haptic feedback from manipulation of the robot. Each subject was told that he/she would be manipulating “objects”. After the subject was properly positioned, the handle of the manipulandum was placed in the hand by the experimenter, in a position that followed that of the start target.

Subjects were first familiarized with the robot and the speed requirements of the task by completing 24 movement trials in the absence of a force field (null field). All subjects then were told that the task would be completed with a new object. Subjects completed three blocks of 180 movements. Each trial was a movement from the start target to one of the three targets, or a movement from one of the three targets to the start target. Subjects were thus required to move in one of six directions, along three axes of movement. The axis was randomized every two trials (out and back to the start). The first and third blocks of movements were completed in a CWFF and the intervening block of movements (the second block) was completed in a CCWFF. The experiment thus followed the A1-B-A2 paradigm. Between each block, subjects rested for five minutes in a separate room.

**Figure 2:**
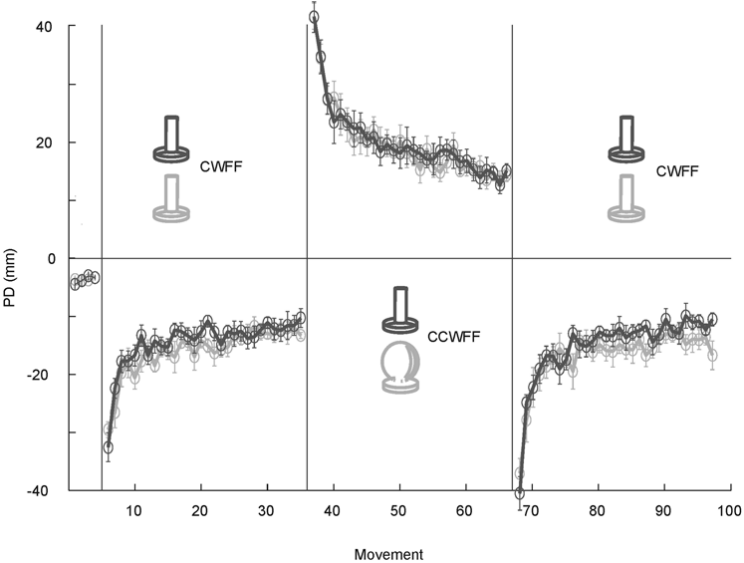
Movement perpendicular distance is shown over the course of movements in the CWFF, CCWFF and CWFF. Data plotted in dark grey represent subjects who grasped the same handle in all three sessions. Data plotted in light grey represent subjects who grasped a given handle shape for the CWFF and a different handle shape for the CCWFF. Each data point represents the mean perpendicular distance over 6 movements, averaged over subjects.

Subjects were randomly assigned to one of two groups. Seven subjects performed the task while the handle of the manipulandum remained the same (a cylindrical handle). For another group of 7 subjects, each force field was associated with a differently shaped handle. The CWFF was transmitted through the cylindrical handle and the CCWFF was transmitted through a spherical handle (see Figure 2). The cylindrical handle was 88 mm tall, 24 mm wide and 84 mm in circumference. These dimensions are in line with those of a previous study using a robotic manipulandum [18]. The spherical handle measured 88 mm in height, 78 mm in width and 250 mm in circumference. Subjects were instructed to use the same grip for both handles, with full contact between the glabrous skin (of the fingers and palm) and the handle. The configuration of the arm was also held constant. To ensure that our results were not due to the particular handle shape, the experiment was repeated using two other groups of subjects, for whom the association between handles and force fields was reversed. Handle shape was counterbalanced in these two groups in order to rule out the possibility that any effects due to handle shape are an idiosyncrasy of the order in which the handles are presented. One group (n = 5) grasped only the spherical handle for all three force fields, and the other group (n = 5) grasped the spherical handle when exposed to the CWFF and the cylindrical handle when exposed to the intervening CCWFF.

Another 7 subjects were assigned to a group in which all three blocks of movement trials were completed in the CWFF, while grasping the cylindrical handle. This group was conceived as a means to investigate how subjects retain their learning of a single force field. By comparing performance in this group with performance in the A1-B-A2 paradigm we can also explicitly demonstrate the interfering effects of the intervening force field (B).

### Data Analysis

Performance was characterized by measuring the curvature of each movement trajectory using perpendicular distance (PD), the maximum orthogonal deviation between the hand and the line segment connecting the start position and the target [12], [17]–[19]. PD reflects a subject's skill in adapting to the force field. The extent of learning was measured not only by observing changes in PD but also by observing performance during catch trials when the force field was suddenly and unexpectedly removed. Each block contained 15 catch trials, scattered throughout the block. Catch trials during force field learning result in perturbed movements called “after-effects”. When first adapting to a force field, catch trials result in no after-effects [1] and as adaptation progresses, after-effects appear on catch trials. In the case of CWFF or CCWFF training, after-effects look like mirror images of the perturbed hand paths seen during early exposure to the force field [12]. Catch trials show that adaptation to novel dynamics is marked by movements that precisely counteract the force field. The steadily increasing magnitude of after-effects indicates the learning of novel dynamics [1]. To ensure that the present results were not an artifact of the chosen dependent measure (PD), we also computed a second measure of movement curvature, the area enclosed by the path of the hand (AREA) from movement start to movement end. Movement start was defined as the point at which hand velocity first reached 10 cm/s; movement end was defined as the point at which hand velocity first dropped below 10 cm/s. For all tests presented below, similar patterns were observed for PD and AREA measures.

Individual PD and AREA scores were collapsed across bins of six movements, and differences between group means were tested using analysis of variance (ANOVA) and Tukey post hoc tests. Data analyses were carried out using custom software routines in Matlab (The Mathworks, Natick MA).

## Results

### Force Field Adaptation

Differences between groups' PD scores were tested using an ANOVA, in which 3 factors were included. Assignment to either the control group (in which handle shape remained constant) or the experimental group (in which handle shape varied with the force field) was included as a between-subjects factor. The subjects' movements over time comprised a within-subjects factor. A third factor was included to address the issue of counterbalancing in the experiment. Recall that handle shape was counterbalanced in the experimental and control groups. In one case, the control group grasped only the spherical handle for all three force fields and the experimental group grasped the spherical handle when exposed to the CWFF and the cylindrical handle when exposed to the intervening CCWFF. In another case, the control group grasped only the cylindrical handle, while the experimental group switched to the spherical handle during the CCWFF. To examine whether or not the assignment of a given handle shape to a force field was of any consequence, counterbalancing was included as a between-subjects factor. It was found that the main effect of counterbalancing and all interaction effects involving counterbalancing failed to reach significance (p>.05 in all cases), indicating that the assignment of handle shape to either CWFF or CCWFF did not affect PD. In the following analyses, counterbalancing was ignored and groups were combined, leaving only one control group and one experimental group.

All subjects were able to adapt to the force fields. Figure 2 shows that initial exposure to the CWFF resulted in curved movements. For both the control group and experimental group, mean PD over the first 12 trials was significantly higher than mean PD in null training (Control F(1,78) = 96.3; Experimental F(1,78) = 99.7, p<.001 in both cases). With training, subjects decreased movement curvature. For both control and experimental groups, mean PD over the last 12 trials was significantly lower than in the first 12 trials (Control F(1,78) = 24.4, Experimental F(1,78) = 23.8, p<.001 in both cases). Similarly, subjects in control and experimental groups were able to adapt to the CCWFF. Mean PD over the last 12 trials was significantly lower than in the first 12 trials (Control: F(1,78) = 50.6; Experimental: F(1,78) = 62.1; p<.001 in both cases). Likewise for the second CWFF, both control and experimental groups showed a significant decrease in mean PD over the last 12 trials compared to the first 12 trials (Control: F(1,78) = 48.6; Experimental: F(1,78) = 32.9; p<.001 in both cases). Table 1 gives means and standard deviations of PD for all conditions.

**Table 1:** Mean perpendicular distance (mm) for null field movements and for initial and final performance in each force field, for subjects who grasped the same handle in all force fields and those who grasped a different handle for each force field.

Force Field	Movements	Control Group Same Handle	Experimental Group Different Handles
Null	1–12	4.4 (1.9)	5.2 (3.0)
CWFF (A1)	initial 12	−27.2 (4.4)	−27.3 (5.2)
	final 12	−11.3 (3.5)	−11.6 (2.9)
CCWFF(B)	initial 12	36.7 (5.8)	37.9 (6.7)
	final 12	13.7 (3.6)	13.4 (3.3)
CWFF(A2)	initial 12	−32.8 (7.2)	−31.0 (6.6)
	final 12	−10.8 (1.9)	−12.8 (5.3)

Values in parentheses indicate one standard deviation.

**Figure 3:**
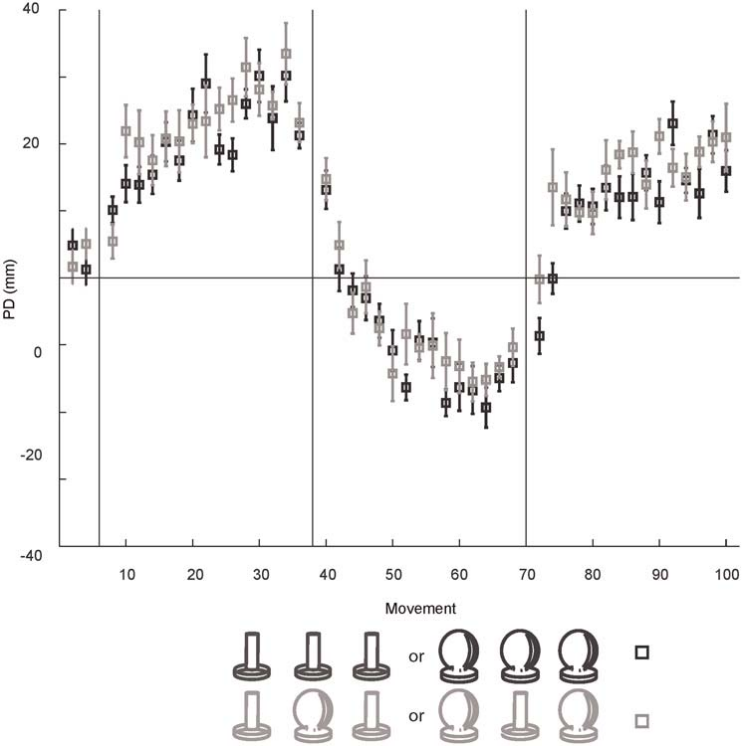
Catch-trial performance in the CWFF, CCWFF and CWFF for subjects who grasped the same handle in all three sessions (dark grey) and subjects who grasped a different handle for each force field (light grey). Each data point represents mean perpendicular distance averaged over subjects for single movements.

Catch trial data also showed that adaptation to the force fields took place, as each field was characterized by after-effects that steadily increased in magnitude as a function of training and opposed the direction of the force field (see Figure 3 and Table 2). For both control and experimental groups, after-effects during initial exposure to the CWFF were relatively small, whereas after-effects at the end of the block were large, and were significantly larger than those at the beginning (Control F(1,65) = 6.9, p<.05; Experimental F(1,65) = 28.2, p<.01). The same pattern was also observed in the other two blocks of force field training (see Table 2).

**Table 2:** Mean perpendicular distance (mm) for catch-trials in each force field, for subjects who grasped the same handle in all force fields and those who grasped a different handle for each force field.

Force Field	Catch-trial	Control Group Same Handle	Experimental Group Different Handles
CWFF (A1)	first	10.1 (6.9)	5.4 (8.4)
	last	21.3 (6.5)	23.2 (9.6)
CCWFF (B)	first	13.2 (9.9)	14.7 (10.5)
	last	−12.7 (10.1)	−10.4 (9.2)
CWFF (A2)	first	−8.7 (9.3)	−0.0 (11.8)
	last	16.0 (10.7)	21.0 (16.7)

Values in parentheses indicate one standard deviation.

Patterns of adaptation did not differ between control and experimental groups. Mean PD over the first 12 trials in the first exposure to the CWFF did not differ significantly between the two groups (F(1,78) = .02, p>.05). Performance during the null field was also similar, ruling out the possibility of a pre-existing difference in the ability to generate straight movements while grasping the robot (F(1,78) = .10, p>.05). Finally, mean PD over the last 12 trials in the CWFF did not differ significantly between the two groups, indicating an equal extent of adaptation in the two groups (F(1,78) = .01, p>.05).

Catch trial data also suggest that adaptation did not differ between the two groups. Mean PD of the first catch trial for the Control Group did not differ significantly from that of the Experimental Group (F(1,65) = 1.0, p>.05). In addition mean PD of the last catch trial for the Control Group did not differ significantly from that of the Experimental Group (F(1,65) = 2.7, p>.05).

### Interference

#### Force Field Trials

A decrement in performance was seen in both Control and Experimental groups when subjects were faced with the CCWFF and subsequent retraining in the CWFF. During early exposure to the CCWFF, PD was greater than that observed during early exposure to the CWFF (see Figure 2 and Table 1). For the Control Group PD was significantly greater during the first 12 trials in the CCWFF than in the CWFF (F(1,78) = 8.2, p<.01). In addition mean PD over the first 12 trials in the CCWFF was significantly higher than mean PD over the last 12 trials of the preceding CWFF (F(1,78) = 60.8, p<.001). For the Experimental group, the data also suggested a decrement in performance upon exposure to the CCWFF. Mean PD over the first 12 CCWFF trials was significantly greater than the corresponding trials in the preceding CWFF (F(1,78) = 12.1, p<.05) and the final twelve trials in the same CWFF (F(1,78) = 69.9, p<.001).

A decrement in performance was also observed during retraining in the CWFF. Notwithstanding previous training in the CWFF, retraining was characterized by performance that was no better than that observed during initial training in the CWFF. In the case of the Control Group, any differences in mean PD over the first 12 trials of the first and second CWFF were not statistically reliable (F(1,78) = 3.1, p>.05). Similarly, for the Experimental Group, differences between mean PD over the first 12 trials of the first and second CWFF failed to reach statistical significance (F(1,50) = 1.3, p>.05).

**Figure 4:**
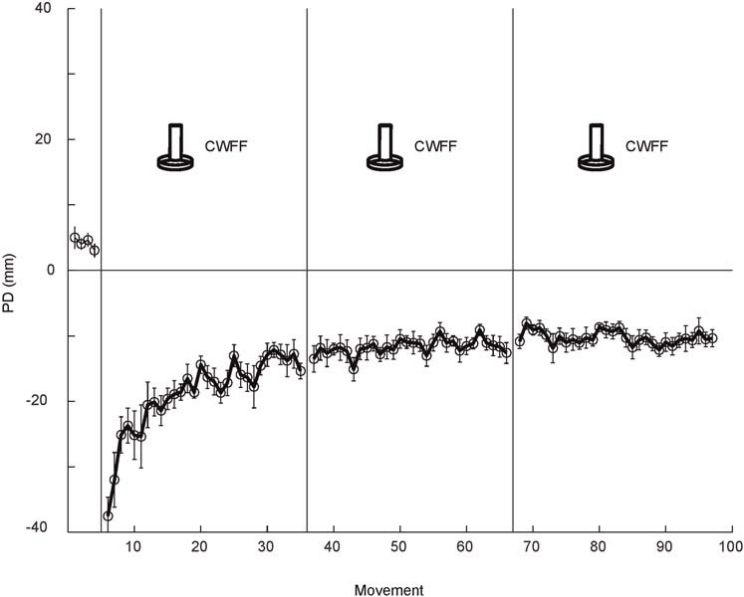
Movement perpendicular distance for subjects who grasped the same handle in three consecutive blocks of the CWFF. Each data point represents the mean perpendicular distance over 6 movements, averaged over subjects.

The performance of control and experimental groups during the second CWFF is a marked departure from that of the group that trained exclusively in the CWFF (see Figure 4). In this retention control group, each of the three blocks of movement trials was performed in the CWFF. The point in the experiment at which subjects in the other groups encountered the second CWFF corresponds to a point at which retention control subjects began their third block of training in the CWFF. Their performance during the early portion of this block surpasses that of the other two groups – mean PD over the first 12 trials of this block was significantly lower than that of the control and experimental groups during their retraining in the CWFF (F(1,78) = 38.7, p<.01). It should be noted that this comparison includes the retention control group's third exposure to the CWFF; in the case of the other groups, training in the CWFF occurred only twice. When the performance of the control and experimental groups during their second exposure in the CWFF is compared to the performance of the retention control during their second exposure to the CWFF (which, for them, is their second block of movement trials), the retention control group is again found to be superior. During the retention control group's second exposure to the CWFF, mean PD over the first 12 trials of this block was significantly lower than that of the control and experimental groups during their retraining in the CWFF (F(1,78) = 27.5, p<.01). Following a full block of training in the CWFF, the movements of the retention control group were less perturbed during subsequent retraining in the CWFF, indicating retention of learning. These findings lend credence to the notion that the experimental and control groups showed interference during retraining in the CWFF.

#### Catch Trials

Catch trial data also showed a decrement in performance in the CCWFF and retraining in the CWFF (see Figure 3). As described above, the progressively increasing magnitude of after-effects indicates that both groups learned to compensate for the force fields. However, the initial direction of after-effects during training in the CCWFF and retraining in the CWFF more closely followed that of the preceding training session. This was observed in both groups. Figure 3 depicts PD in the catch trials as a function of training in all three blocks. PD was measured in both the opposite direction of the force field (the anticipated direction of after-effects) and in the same direction as the force field.

For the Control Group, mean PD in the first catch trial of the CCWFF did not differ significantly from the last catch trial in the preceding CWFF (F(1,65) = 2.9, p>.05). This shows that the after-effects observed during early training in the CCWFF were in the same direction of the force field and thus similar to catch trials in the preceding block, which were also characterized by after-effects in the counter-clockwise direction. This does not match the catch trial performance in the preceding force field. In the first CWFF block, after-effects were chiefly in the opposite direction to the force field, whereas the first catch trials in the CCWFF were in the same direction of the force field (see Figure 3). A similar pattern was seen for the Experimental Group. Mean PD in the first catch trial of the CCWFF did not differ significantly from the last catch trials in the preceding CWFF (F(1,65) = 3.4, p>.05). Again, this does not match the catch trial performance in the preceding force field, where after-effects opposed the direction of the force field.

The pattern of catch trials during the CCWFF, specifically, that the initial direction of after-effects more closely followed that of the preceding training session, was also observed during the second CWFF. Here the early catch trials were characterized by after-effects that were in the clockwise direction (see Figure 3). Mean PD in the first catch trial of the second CWFF did not differ significantly from the last catch trial in the CCWFF, for both the Control Group (F(1,65) = 3.9, p>.05) and the Experimental Group (F(1,65) = 3.9, p>.05).

#### Comparison Between Groups

While both groups showed evidence of interference, there was no difference between control and experimental groups in the amount of interference. No significant difference was observed between groups in mean PD over the first 12 trials in the CCWFF (F(1,78) = .22, p>.05). In addition no significant difference was observed between groups in mean PD over the last 12 trials in the CCWFF (F(1,50) = 0.13, p>.05). This shows that the two groups did not differ in their ability to adapt to the CCWFF. Performance during retraining in the CWFF was similar across the two groups. No significant difference was observed between groups in mean PD over the first 12 trials in the second CWFF (F(1,50) = 0.10, p>.05). Likewise, no significant difference was observed between groups in mean PD over the last 12 trials in the second CWFF (F(1,78) = .07, p>.05), showing that the two groups did not differ in their ability to adapt to the second CWFF.

Similarly, no differences were observed between control and experimental groups in catch trial performance. Mean PD for the first catch trial was not significantly different for the Experimental versus Control groups (F(1,65) = .28, p>.05). Likewise, in the second CWFF mean PD for the first catch trial was not significantly different for the two groups (F(1,65) = 2.5, p>.05).

#### Believers vs Non-Believers

**Figure 5:**
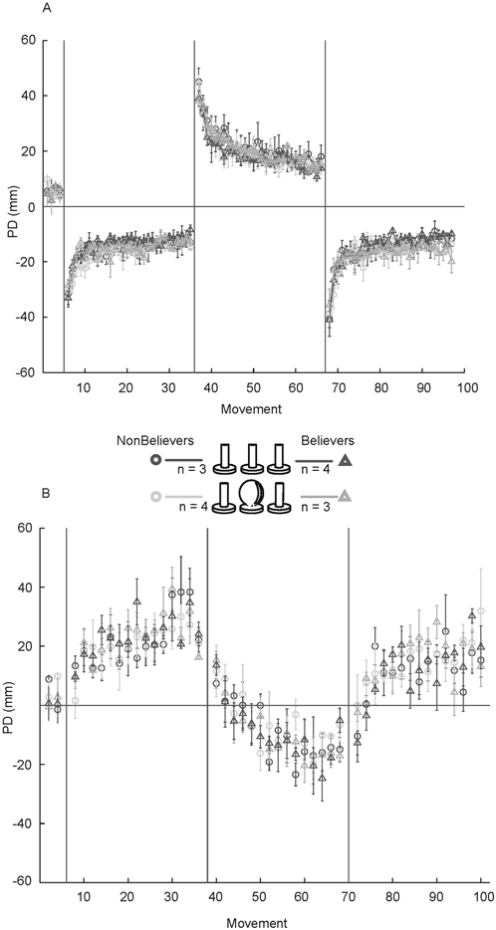
Believers versus non-believers. a: Mean perpendicular distance for movements in the CWFF, CCWFF and CWFF are shown for subjects who reported believing that they were grasping two different objects in the CWFF and CCWFF sessions (“believers”, indicated by traces marked with triangles), and those who reported believing that they were grasping the same object in all sessions (“non-believers”, indicated by traces marked with open circles). For each group data are further decomposed into subjects who grasped the same cylindrical handle for all three sessions (dark traces), and those who grasped the cylindrical handle for CWFF sessions and a spherical handle for the CCWFF session (light traces). Each data point represents the mean perpendicular distance over 6 movements, averaged over subjects. b: Catch-trial performance for believers and non-believers. Each data point represents mean perpendicular distance averaged over subjects for single movements.

The goal of this study was to assess whether the addition of distinct haptic cues associated with different force-fields would promote the independent acquisition of different internal models, and correspondingly, whether a reduction in interference would result. One possibility is that no statistically reliable differences were observed between control and experimental conditions, because of inter-subject variability in the extent to which the haptic cues were successfully integrated by the motor system. To test this possibility at an explicit level, we interviewed subjects at the end of the experiment to assess their subjective impression of the nature of the motor learning tasks. Each subject was asked “Did you have the impression that you were handling different objects in each session, or did you believe you were handling the same object, which was behaving differently in each session?” We then partitioned both the control and experimental groups according to their responses – “believers” who believed they were handling different objects (control group n = 4, experimental group n = 3), and “non-believers”, who had the impression that they were handling a single object (Control group n = 3, experimental group n = 4). We repeated all of the statistical tests reported above, and in all cases the same patterns were observed. In no case were statistically significant differences observed between “believers” and “non-believers” (see Figure 5).

#### Statistical Power

To measure statistical power in the analyses, an estimate of effect size was used based on previous studies of motor learning [10], [20] in which sample size and number of trials was roughly equivalent to the present study. A reduction in interference was observed in these previous studies, as a result of other experimental manipulations not related to the current study. Power in the present experiment is estimated to be above 0.90. Moreover, visual inspection of Figures 2, 3 and 4 suggests that there is no trend of a reduction in interference. Performance in the motor task appears equal across groups. When reporting differences in means that fail to reach statistical significance, it is crucial to ensure that the results did not arise as a consequence of low statistical power. In the present study, it is unlikely that the analyses failed to detect real differences between the experimental and control groups, given the apparent similarity in performance across groups and high estimated statistical power.

## Discussion

We found that pairing two opposing force fields with distinct haptic cues associated with grasp did not lead to a reduction in interference. While subjects learned to counteract both force fields (this was revealed in subjects' ability to reduce movement curvature, as well as in the increasing magnitude of after-effects during catch trials over the course of training), the same magnitude of interference was observed in both control and experimental groups. Following adaptation to a CWFF, training in a CCWFF was marked by a decrement in performance relative to that in the initial CWFF. The same decrease in performance was observed in both groups. Retraining in the CWFF was also met with difficulty. Performance was no better than that during initial CWFF training. Again, the same pattern was observed for both control and experimental groups. Regardless of subjects' reported beliefs about whether the two force fields (and in the case of experimental group subjects, the two handle shapes) corresponded to distinct objects, the same magnitude of interference was observed. This suggests that in the learning paradigm tested here, haptic cues on their own are not sufficient for the learning of two different force fields.

The role of haptic cues in force field learning has received relatively little attention. In a recent study, interference was examined during the learning of two equal and opposite force fields, one of which was applied to the hand and the other directly to the arm [9]. As in the present study, the two force fields were associated with different sensory cues and interference was not reduced. The researchers argued that the brain does not independently represent loads applied to either the hand or the arm. However, their study was not an explicit simulation of two distinct handheld objects. Hwang and colleagues report that conscious awareness of a force-field perturbation had a small, but significant positive effect on motor skill acquisition [21]. Similarly, Imamizu and colleagues showed that explicit prior cognitive knowledge about the nature of visuomotor rotations facilitated the ability of subjects to switch between two opposing rotations, and also improved asymptotic performance [22]. Previous studies suggest that force field learning may reflect the acquisition of a neural representation of a grasped object [14], [15] and so may share some features with object manipulation. Guided by these recent findings, the present study is a systematic examination of the effectiveness of haptic cues in promoting learning and retention of two motor skills.

Persistent interference during the learning of two equal and opposite force fields has been observed in previous studies using similar A1-B-A2 paradigms and the findings of the present study are consistent with these [7], [8], [12]. However, the specific nature of the impediment is not entirely clear. In particular, it is unknown if the observed impediments to learning were the result of both proactive and retroactive interference, or proactive interference alone. When training in one field is set amid training sessions in the other field, performance in both fields is impeded. The first training session (A1) is followed by hindered performance in the second intervening training session (B). It is most likely that these impairments are due to an effect of previous learning (proactive interference). In previous studies it has been argued that a neural representation of initial learning persists and inhibits performance during subsequent learning [4], [12], [23]. In the present study, following the second, intervening training session performance in the third training session (A2) was also negatively affected. Despite having learned the force field previously during A1, retraining in A2 was marked by performance at the level of naïves, or worse. The intervening field seemingly led to a complete unlearning of the initial field. Poor performance in A2 can be attributed to proactive interference due to previous learning in B. Alternatively, poor performance in A2 can be attributed to retroactive interference, in which the learning of B interferes with previous learning (namely, A1), disrupting skilled performance when field A is introduced a second time during A2.

The introduction of “washout” periods in which subjects train in the absence of a perturbation appears to be relevant for studies of force field learning. When subjects adapt to a perturbation (A) and then to a counter-perturbation (B), a washout period between the two training sessions is thought to prevent proactive interference [8], [10]. Previously, it has been demonstrated that even with washout periods in place, performance during A2 is no better than that during initial training in A1, indicating that the effect of an intervening training session, B, is retroactive interference [8]. Washout periods were not used in the current study as they are at odds with the spirit of the experimental design, which was in part to simulate the manipulation of two distinct objects. The progressively increasing magnitude of after-effects indicates that both groups learned to compensate for the force fields. However, the initial direction of after-effects during training in the CCWFF (B) and retraining in the CWFF (A2) matched that of the preceding training session. This pattern of after-effects is consistent with the notion that poor performance during both the CCWFF and the second CWFF block were due to proactive interference. It should be noted that the present results are not inconsistent with the idea that both proactive and retroactive interference contribute to deficits in performance during retraining in the CWFF.

The causes of interference and the question of whether interference can be attenuated remain controversial. Several candidate explanations of interference have been proposed. Previous studies of interference explored the idea that introducing a delay between training in A1 and B may ameliorate interference. These studies tested the possibility that motor learning undergoes a process of consolidation, whereby memories become resistant to interference with the passage of time (for a review of consolidation and interference in a broader context, see [24]). It has been asserted that a 4- or 5-hour delay between initial training in a force field and training in an equal and opposite force field reduces interference, and that during this time motor skill learning undergoes consolidation [7], [12]. However, Caithness and colleagues showed in a rigorous set of experiments that interference is observed regardless of the length of the delay separating training sessions [8]. Krakauer and colleagues argued that the learning of visuomotor rotations undergoes consolidation, as long as initial training in task A is extensive, there is a sufficiently long delay between tasks A and B, and there are washout periods between tasks [10]. Clearly, there are a number of unresolved issues surrounding the idea of consolidation in force field learning.

Another potential explanation of humans' poor ability to learn two motor skills focuses on the issue of task similarity. The learning of one motor task is thought to generalize to similar tasks [1], [17], [25]–[30]. When successful completion of a second task requires a strategy or action that differs from the first, generalization is detrimental [31]. This sort of generalization could be the basis of interference in motor skill learning. One possibility is that interference is caused by a lack of informative cues to signal the requirements of two motor tasks, such as two force fields. Several different contextual cues have been explored, including joint configuration [25], visual signals [32], [33], and using different effectors for each motor task [31].

The present experiment was a principled examination of haptic feedback on its own. Our results suggest that haptic cues alone are not sufficient for the learning of two force fields. However this does not preclude the possibility that haptics are important cues when associated with other object-related and environmental signals. In this study all other contextual cues such as arm posture, the training environment and the movement task remained identical, and in that sense the learning of both the CWFF and the CCWFF remained very similar. It is possible that haptic cues aid in the learning of two force fields if they are accompanied by a host of additional contextual cues that normally follow switching between motor tasks that are less similar. Previous studies that relate force field learning to object manipulation [14], [15] show that force field learning does not generalize to movements made in free space. It is possible that release of the robotic device involves the removal of subtle dynamic cues inherent to the device and that this may provide an additional signal to aid in switching between motor tasks, namely, force field learning and homologous reaching movements in free space. Recent studies have shown that interference when learning two force fields is at a maximum when the direction of applied forces in one field opposes the other [34], [35], as in the present study. This raises the possibility that the effectiveness of haptic cues on their own may be limited in the present study by the relatively challenging task of learning two opposite force fields.

One possibility is that haptic cues on their own are not sufficient for the learning of multiple force fields because haptic cues are not directly informative in terms of dynamics. Lederman and Klatzky found that when vision of objects is obscured, subjects engage in manipulative actions with the hands called “exploratory procedures” or EPs to ascertain defining features of the object [36]. Different EPs communicate different features of grasped objects. For example lateral motion of the fingers across an object's surface chiefly provides information regarding texture. EPs vary in terms of the breadth of information they provide. Some EPs yield narrow information while others are broader in terms of their informativeness. Lederman and Klatzky found that the sequence of EPs always begins with a grasp followed by a lift [36]. While these two EPs provide the most coarse information, dynamically they communicate little, aside from texture and weight, both of which could be afforded by unsupported holding. Without clear vision of grasped objects, EPs are most effective for providing the nervous system with information regarding the surface properties of objects. Unimpeded vision is optimal for providing the nervous system with information regarding the spatial and geometric features of objects [36] and perhaps information about dynamics [18]. The present experiment did not entail lifting as movements were restricted to the horizontal plane, and so it could be argued that haptic cues alone could not provide any direct information about dynamics.

Another possibility is that the sort of haptic cues tested here could facilitate learning opposite force fields, but may require more extensive training. In one way, the A-B-A paradigm tested here is essentially a single-trial learning paradigm, because subjects are only exposed to a single transition between fields and handles. Given that subjects are required to learn not only two different force fields, but also how to switch between them given an arbitrary cue (handle shape), more extensive training including a greater number of switches between force fields may be required to successfully learn to associate handle shape with force field direction. Indeed, a number of studies have shown that given extensive training, subjects can learn to use contextual cues such as color to switch predictively between two motor skills [22], [32], [33].

The inability of subjects to learn both force fields in the present experiment may be due to a number of factors specific to curl fields. First, when learning to manipulate grasped objects in more naturalistic settings the arm is not restricted to a horizontal plane and objects are more akin to simple inertial loads. In the case of different grasped objects, the associated dynamics are typically not in direct opposition to one another as they are in the curl fields used here. Moreover the direction of imposed forces when moving in a curl field is perpendicular to the direction of hand motion. In the case of inertial loads forces are along the movement direction of the hand. Curl fields are dependent on the velocity of the hand, while inertial loads are dependent on acceleration. Curl fields also differ from inertial loads in terms of kinematics and resulting movement errors. Curl fields produce curved movement trajectories, while grasping and lifting unfamiliar objects do not alter the direction of movement trajectories in the same dramatic manner. Finally forces are applied in two opposite directions in an inertial load - initial acceleration is met with a force that opposes motion and deceleration is met with a force that assists motion, or rather, resists deceleration. These forces are commensurate with hand acceleration and the mass of the grasped object. In the case of curl fields, forces are applied in only one direction, with a magnitude that is commensurate with hand velocity only.

Future studies of interference in motor learning may focus on associating two force fields with a combination of cues, including haptic cues. Additionally, future studies may further probe the question of whether or not haptics is diagnostic of dynamics by using different motor paradigms in which the relationship between haptic cues and dynamic properties is experimentally manipulated.
